# metaSNV: A tool for metagenomic strain level analysis

**DOI:** 10.1371/journal.pone.0182392

**Published:** 2017-07-28

**Authors:** Paul Igor Costea, Robin Munch, Luis Pedro Coelho, Lucas Paoli, Shinichi Sunagawa, Peer Bork

**Affiliations:** 1 Structural and Computational Biology Unit, European Molecular Biology Laboratory, Heidelberg, Germany; 2 Department of Biology, Institute of Microbiology, ETH Zurich, Zurich, Switzerland; 3 Department of Biology, Ecole normale supérieure, Paris, France; 4 Max-Delbrück-Centre for Molecular Medicine, Berlin, Germany; 5 Department of Bioinformatics, Biocenter, University of Würzburg, Würzburg, Germany; 6 Molecular Medicine Partnership Unit, Heidelberg, Germany; Columbia University Medical Center, UNITED STATES

## Abstract

We present metaSNV, a tool for single nucleotide variant (SNV) analysis in metagenomic samples, capable of comparing populations of thousands of bacterial and archaeal species. The tool uses as input nucleotide sequence alignments to reference genomes in standard SAM/BAM format, performs SNV calling for individual samples and across the whole data set, and generates various statistics for individual species including allele frequencies and nucleotide diversity per sample as well as distances and fixation indices across samples. Using published data from 676 metagenomic samples of different sites in the oral cavity, we show that the results of metaSNV are comparable to those of MIDAS, an alternative implementation for metagenomic SNV analysis, while data processing is faster and has a smaller storage footprint. Moreover, we implement a set of distance measures that allow the comparison of genomic variation across metagenomic samples and delineate sample-specific variants to enable the tracking of specific strain populations over time. The implementation of metaSNV is available at: http://metasnv.embl.de/.

## Introduction

Recently, strain-level analysis of metagenomes has been shown to be feasible even for complex communities such as the human gut [1] and a number of tools have been consequently developed to enable researchers to study microbial communities at this level of resolution. These tools differ considerably in approach and assumptions as well as in the type of information they provide as output. As such, conspecific strains can be disentangled based either on gene content [2,3] or using specific SNVs [1,4]. The latter approach is the category that the current work falls under, though here also the specifics vary, with some tools attempting to reconstruct mini-haplotypes, based either of core species genes [5] or species-specific marker genes [6], while others try to characterize the genome-wide variation landscape, without endeavouring to construct haplotypes [1,4]. All these approaches are dependent of the availability of reference genomes and may thus only be applied to well characterized environments. Complementary methodology is being developed to tackle the challenge of characterizing samples for which few or no reference genomes are available, by combining metagenomic assembly with single cell sequencing [7].

There are two main challenges in the use of these of the reference dependent tools, which are of interest here: usability and interpretability. For the former, as the number of samples to compare increases, considerations such as run-time and storage footprint become increasingly important. In the case of the latter, the tools currently available output primary analyses that require additional work to interpret the results.

Here, we present a fast and scalable tool, metaSNV, for quantifying genomic variation based on original concepts and procedures of Schloissnig et al. [1], with additional functionality and packaged as an easy to use pipeline. We compare its performance and output to MIDAS as an alternative implementation [4], which also aims at characterizing whole genome variation based on mapping to one representative genome per species. We do not perform a comparison to the output of tools that use only a subset of the genome to determine strain haplotypes, be it a set of common marker genes [5] or a species-specific set [6].

metaSNV uses a collection of microbial reference genomes, where each species is represented by a single representative genome to avoid redundancy [8,9]. Alternatively, users may specify their own reference genome or gene collection. We show that our approach identifies extensive variation within microbial species and that this variation is informative in quantifying differences between metagenomic samples. Towards this end, metaSNV also implements a set of distance measures that can be used to compare the variation profiles between samples in order to determine genetic distances of strain populations and to identify relations to explanatory variables of interest (sampling site, environmental conditions, health states, etc.).

As a demonstration, using data from the Human Microbiome Project (HMP) [10], we show that the genomic variation of most bacteria that inhabit the human oral cavity is highly correlated with the specific sub-habitat that they have been collected from (e.g. tongue dorsum vs. supra-gingival plaque) and that individual SNV profiles are stable over time.

## Materials and methods

**Fig 1:**
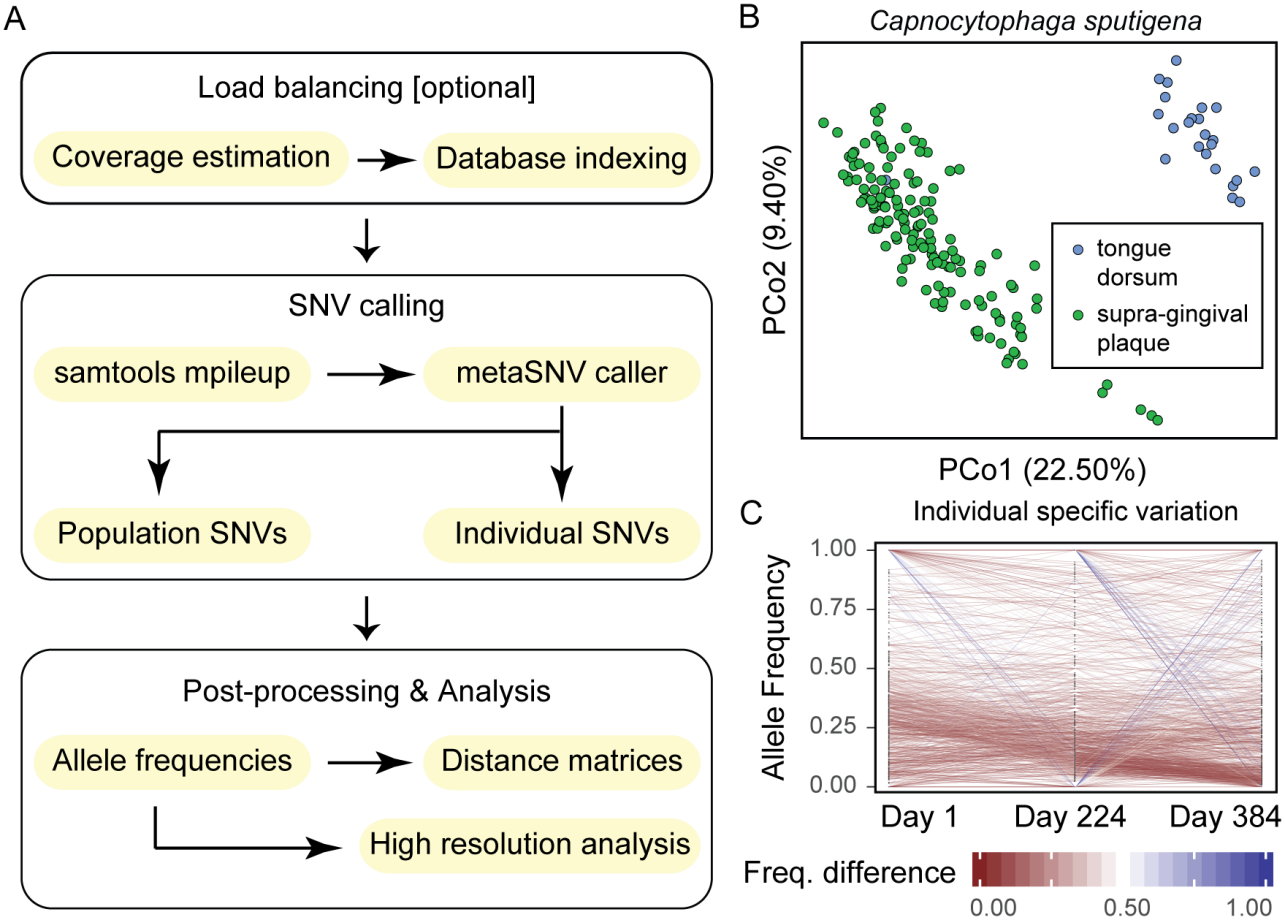
Overview of analysis pipeline and example results. (A) shows the SNV calling and analysis workflow, consisting of an optional pre-processing step, which splits the computation load into subsets of similar size based on the genome coverage, the main SNV calling step and further post-processing of the raw output, which can be tailored according to the aim of the analysis. (B) shows the Principal Coordinate Analysis projection of a pairwise distance between oral samples, based on population SNVs, which clearly separates strain populations in tongue dorsum samples from those in supra-gingival plaque samples. (C) shows the tracking of the individual SNV frequencies within an individual over a period of 384 days. Each line represents one variant position and the respective colour encodes the amount by which the allele frequency of that position changed over time; red represents stable variants that maintain their frequency while in blue are positions which dramatically change their frequency in the population. Only a small number of positions vary over the measured period, with most remaining at approximately the same population frequency, suggesting great stability of strain populations within the individual.

The pipeline input is a list of alignment files in SAM/BAM format, which contain the results of mapping metagenomic samples to a reference genome database. Results presented here were computed using bwa as an aligner [11]; however other tools can be used. In particular, we describe the parameters we used to quality control metagenomic sequences on the tutorial webpage (http://metasnv.embl.de/) and how to use bwa and Ngless (http://ngless.embl.de/) to produce BAM files [12] which can be used as input for metaSNV. As previously stated, the reference genome database may be a custom one created by the user or the one deployed with the current software [9]. metaSNV is structured as a sequence of three processing steps (Fig 1A), with the first two wrapped together in one command and a separate script for post-processing. Firstly, we determine the average coverage over each reference genome in each sample. For this, we run qaCompute per sample (a tool from the qaTools suite https://github.com/CosteaPaul/qaTools, which is deployed with metaSNV) and aggregate the coverage information. This step can be parallelized as each sample coverage estimation is independent of all others. In the next step we compute the genomic variation and output all of the variant positions that meet the imposed quality criteria. Here we take advantage of the mpileup tool in samtools [13], in order to obtain per-position variant information. These variant calls then get filtered based on the given criteria and if a gene position file is given, get annotated as synonymous or non-synonymous change compared to the reference allele. For this processing steps also, metaSNV supports multi-threading to use multiple cores. Lastly, we provide post-processing analyses of the SNV landscape and allow the user to compute per species pair-wise distance matrices of samples, as well as evolutionary measures such as nucleotide diversity and fixation index [14,15].

If desired, metaSNV can automatically estimate a balanced split of the input data and divide the overall work into multiple jobs, which can then be executed in parallel over many machines in a high-performance computing cluster.

### Genome coverage estimation

Given an alignment file (in standard BAM format [13]), we estimate the vertical coverage, that is the number of bases covering each genome divided by its length. We also compute the horizontal coverage, namely the percentage of the genome that is covered by at least one read. Based on the coverage information, the reference may be divided into parts that are estimated to require similar execution times.

### Population and individual SNV calling

We determine the existence of a candidate variant on a per-nucleotide basis, building upon the mpileup tool in the samtools package [13]. All reads from all samples that align to a given position are considered together. If at least four variant containing reads cover a position (across all samples), it is considered a potential SNV [1]. Variants are split into two classes: population and individual variants. The former are non-reference nucleotides observed in more than 1% of all reads combined across all samples. The individual variants are those that fall below the 1% frequency population threshold, but are confidently observed in at least one sample (at least four reads containing the variant). If multiple different non-reference nucleotides are observed, all are reported independently. We observed such multi-allelic positions to be rare in our experiments: 3.7% of the population variants and 1.6% of the individual ones. While the four reads criterion filters out sequencing errors randomly distributed across the genome, the 1% criterion eliminates random sequencing errors that accumulate in the same position when depth of coverage reaches very high numbers; conservative error rates have been estimated for these cut-offs to be in the range of 0.35–0.7% [1]. The thresholds described are the default settings for the pipeline, but may be customized by the user if desired.

### Post-processing and analysis

Taxon, sample and position filters are applied post SNV calling. Within each sample, we consider a taxon to have been observed if the respective genome has a vertical coverage of at least 5x and a horizontal coverage of at least 40%. We impose the 5x vertical coverage cut-off to prevent ascertainment biases due to spurious coverage. In addition, as high vertical coverages can be reached by spurious mapping of sufficiently high numbers of short reads to highly conserved genes or genomic regions, we additionally impose a horizontal coverage filter. We base the default cut off (40%) on a previously estimated lower bound of the genome percentage shared by distinct *E*. *coli* strains [16]. We note that this lower bound is rather conservative and we generally find more than 80% horizontal coverage at 5x in human faecal samples. However, this ensures the presence of the given species in the sample of interest. Both these cut-offs can be customized by the user, though we recommend using the proposed ones to ensure the accuracy of subsequent distance estimations. Resulting SNVs are further filtered to only consider those positions which were covered at 5x in at least 50% of samples, ensuring that only variation over commonly observed positions is considered. For downstream processing, we implemented a per-taxon computation of pair-wise distance matrices between all samples, based on these filtered SNVs. These distances are based on non-reference allele frequencies across all the pair-wise observed variants. Namely, a Manhattan distance, which adds the absolute frequency difference per site and normalizes to the total number of comparisons. That is, the number of sites for which the comparison was possible; if a position was not observed in a sample, it is ignored in the calculation. Additionally, we offer a “major allele” distance, which only considers differences in the major allele per site; that is, frequency differences greater of equal to 60%. We note that if a position has multiple variants, these are considered independently. Finally, nucleotide diversity (π) [14,17] within and between samples and fixation indexes (F_ST_) [15] can be adapted to metagenomics data [18] and computed for each species as previously described [1]:

$$\pi \left(S_{1},S_{2},G\right)=\frac{1}{G}\overset{n_{SNVs}}{\underset{i=1}{\Sigma }}\sum_{N_{1}\in \left\{ATGC\right\}}x_{S_{1},i,N_{1}}\sum_{N_{2}\in \left\{ATGC\right\}/N_{1}}x_{S_{2},i,N_{2}}$$ (1)

$$F_{ST}=1-\frac{\pi_{within}}{\pi_{between}}=1-\frac{\left(\pi \left(S_{1},S_{1},G\right)+\pi \left(S_{2},S_{2},G\right)\right)/2}{\pi \left(S_{1},S_{2},G\right)}$$ (2)

Where G is the size of the genome, and x_S,i,N_ the frequency of nucleotide N, at position i in the genome, in sample S. All measured described above result in values from 0 to 1, with 1 denoting the greatest dissimilarity between two populations.

## Results and discussion

We have applied the SNV pipeline to 676 shotgun metagenomes from the oral cavity, collected as part of the Human Microbiome Project (HMP) [10]. The result for *Capnocytophaga sputigena* demonstrates that samples from the tongue dorsum of an individual’s oral cavity cluster separately from those collected from supra-gingival plaque (Fig 1B). This result provides strong evidence that the strain populations inhabiting the two habitats are divergent, reminiscent of previously described ecotypes [19,20]. Furthermore, metaSNVs enables the tracking of strains within individuals over time using individual specific variant positions (Fig 1C). Thus, we can track the evolutionary path of SNVs and show that they can be remarkably stable within an individual, even when measured ~400 days later. Specifically, we note that the frequency of the vast majority of variants in the population stays relatively constant, with only few positions being fixed or cleared from the population.

**Fig 2:**
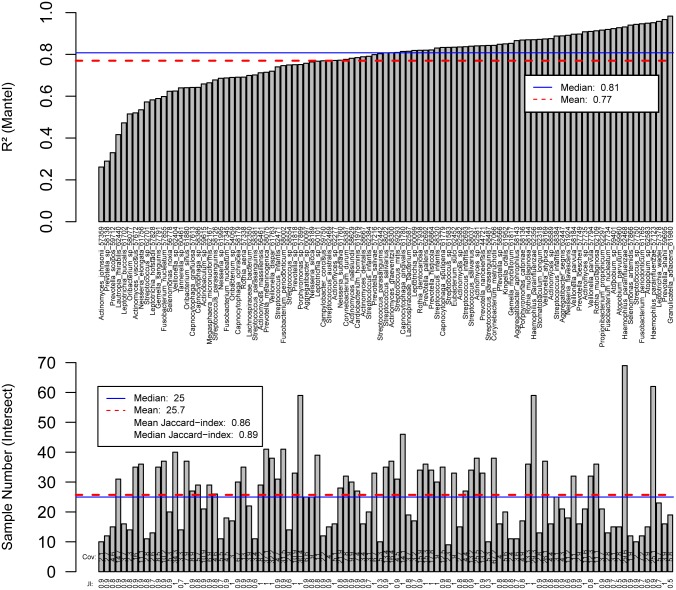
Comparison of metaSNV and MIDAS results. Correlation coefficient (R^2^, mantel) for the pairwise distance matrices generated by MIDAS and metaSNV (top). Compared are only sample intersects for species examined with both methods. Jaccard indices for the sample overlap per species was computed (bottom). The average sample number and average Jaccard index over all samples intersect is shown in the legend.

To compare our results with MIDAS [4], we selected two sites in the oral cavity, tongue dorsum and supra-gingival plaque, and analysed 80 randomly selected HMP samples from these body sites, supra-gingival plaque (N = 40) and tongue dorsum (N = 40). As differences in the called positions themselves are not informative, we computed the Manhattan distance on the allele frequencies using the output from both tools, while running them with similar parameters (using merge_midas.py snps with—min_samples 10—sample_depth 5.0—fract_cov 0.4—site_depth 5—site_prev 0.5—site_maf 0.01 and metaSNV_post.py with -m 10). The distances computed are comparable, with a median R^2^ of 0.81 for the common species and sample intersects, suggesting both methods capture the same genomic variation profile. Common species overlap with a Jaccard-index of 0.86 and the sample intersects per species average a Jaccard-index of 0.89. (Fig 2). Thus, the two methods are able to assess genome variation across the same samples and the resulting characterization is mostly the same.

### Runtime Benchmarking

In order to compare running time and storage footprint we used both approaches with matching parameters and up to 32 CPUs per job. The average runtime for each processing step (alignment, species abundance estimation, SNV calling, filtering and post-processing) was computed by averaging the real time for each individual job (up to 80 jobs per processing step, one for each sample or split). The pipeline's absolute runtime is the sum of all necessary tasks, hence the average real-time to run the total work-flow from fastq files to distance matrix (Table 1).

**Table 1:** Resource comparison for metaSNV and MIDAS.

	Jobs	CPU/Job	CPUs	Max RAM	Time/Job	Time	Disk	CPU Time	Total RAM
	*#*	*#*	*#*	*[GB]*	*[min]*	*[min]*	*[GB]*	*[min]*	*[GB]*
**MetaSNV**									
Alignment	80	32	2560	51.00	100	8000		256000	2484
Species	80	1	80	0.02	20	1600		1600	1.7
SNVs	12	2	24	0.16	100	1200		2400	1.9
Filter/merge	12	1	12	0.02	5	60		60	0.2
Post	1	1	1		1	1		1	0
**TOTAL**	185	37	2677	51.20	226	10861	241	260061	2488
**MIDAS**									
Species	80	32	2560	7.80	50	4000		128000	302
SNVs	80	32	2560	2.50	310	24800		793600	626
Filter/merge	1	1	1	1.60	3094	3094		3094	0.9
Post	1	1	1		1	1		1	0
**TOTAL**	162	66	5122	11.90	3455	31895	537	924695	930
MIDAS/MetaSNV	**0.88**	**1.78**	**1.91**	**0.23**	**15.29**	**2.94**	**2.23**	**3.56**	**0.37**
									From Alignment
MIDAS/MetaSNV	**1.54**	**13.20**	**43.78**	**59.50**	**27.42**	**11.15**	**-**	**227.70**	**242.4**
									From BAM

Breakdown of resource usage, including number of jobs, number of CPUs, maximum and average RAM usage, CPU time and storage footprint (this number does not include the original fasta files used in the analysis).

metaSNV processed all the samples in 226 minutes (132 minutes if the samples were already aligned) and produced 18 GB output (241 GB including the alignment files). This processing time includes alignment (BWA), species abundance estimation (qaCompute), SNV calling (samtools + metaSNV called in parallel computing modus) and post-processing (filtering). In comparison, MIDAS ran for 3455 minutes and produced 537 GB output.

The difference in storage footprint is explained by the fact that metaSNV only outputs positions at which at least one variant across all samples was observed, while MIDAS output all positions. Overall, metaSNV was 15.3 times faster than MIDAS while using 48% less CPUs (2677 total) and has less than half the storage footprint.

### Availability of reference genomes: Limitations and perspectives

metaSNV can be broadly applied to investigate bacterial populations across varying habitats, hosts or clinical conditions. One important consideration, however, is that of availability of reference genomes. At the moment, the collection we provide contains representative genomes for over 5,000 bacterial species [9], though they represent a biased sample of different environments. For instance, the current database only captures a fraction (6%) of the reads collected from the *Tara* Oceans expedition. Nonetheless, the recent release of newly sequenced prokaryotes based on phylogenetic coverage could significantly improve the number of species for previously under-sampled habitats [21]. Additionally, the improvement culture-independent sequencing techniques such as single-cell sequencing or reference-independent approaches [7] could further reduce such biases.

Taken together, we have shown that metaSNV offers a fast, scalable and reliable way of quantifying prokaryotic single nucleotide variation in hundreds of samples. Moreover, we provide easy to use scripts for analyzing this variation in different settings both to compare populations across samples and to track them over time.
